# A longitudinal investigation of school absenteeism and mental health challenges among Canadian children and youth in the COVID-19 context

**DOI:** 10.3389/frcha.2025.1604431

**Published:** 2025-07-31

**Authors:** Amanda Krause, Maria Rogers, Yuanyuan Jiang, Emma A. Climie, Penny Corkum, Janet W. T. Mah, Natasha McBrearty, J. David Smith, Jess Whitley

**Affiliations:** ^1^ School of Psychology, Faculty of Social Sciences, University of Ottawa, Ottawa, ON, Canada; ^2^ Psychology Department, Faculty of Social Sciences, Carleton University, Ottawa, ON, Canada; ^3^ School of Counselling, Psychotherapy, and Spirituality, Saint Paul University, Ottawa, ON, Canada; ^4^ School and Applied Child Psychology, Werklund School of Education, University of Calgary, Calgary, AB, Canada; ^5^ Department of Psychology and Neuroscience, Faculty of Science, Dalhousie University, Halifax, NS, Canada; ^6^ Department of Psychiatry, Faculty of Medicine, University of British Columbia, Vancouver, BC, Canada; ^7^ Faculty of Education, University of Ottawa, Ottawa, ON, Canada

**Keywords:** school absenteeism, externalizing mental health problems, internalizing mental health problems, child and adolescent, longitudinal

## Abstract

School absenteeism across the globe has risen dramatically since the COVID-19 pandemic. Literature indicates that children and youth of all ages are struggling to attend school regularly, leading to problematic outcomes both concurrently and across time. As well, research demonstrates that children and youth who experience mental health challenges are at greater risk of increased school absenteeism rates. The present study investigated the school attendance patterns of Canadian children and youth and the longitudinal and bidirectional links with mental health challenges within the COVID-19 pandemic context. The study sample consisted of 72 children and youth, using parent reports. Parents were asked to complete an online questionnaire which included questions about the demographic characteristics of themselves and their child, their child's school attendance patterns, and their child's mental health challenges. Preliminary descriptive statistics were run in relation to school absenteeism. Two separate path analyses were conducted to determine the longitudinal links between school absenteeism and mental health (split into externalizing and internalizing behaviours) across two timepoints (Time 1 [T1]: Fall 2022, Time 2 [T2]: Spring 2023). These analyses indicated concurrent links between mental health difficulties and school absenteeism. Importantly, path analyses also showed that absenteeism at T1 predicted poorer mental health at T2, indicating that school absenteeism may be one of the driving factors in the causal relationship. A bidirectional effect was found between externalizing behaviours at T1 and absenteeism rates at T2. The reasons for school absenteeism were examined across each time point and for both the externalizing and internalizing groups separately. The present study highlights the complex interplay between mental health and school absenteeism in the context of the COVID-19 pandemic. It provides avenues for effective intervention to better support children and youth struggling with mental health and school absenteeism.

## Introduction

School absenteeism data since the COVID-19 pandemic is telling a compelling and concerning story—school absences, for students of all ages, have increased and have not returned to pre-pandemic levels (1–3). Some experts on the topic have warned against the normalization of school absenteeism since the pandemic (4, 5), describing a possible shift in cultural values around school attendance and education. This shift is concerning given the many known and well-documented links between school absenteeism and concurrent and longitudinal negative outcomes (6, 7). Concern about school absenteeism is a trend that has been observed globally. Indeed, high-quality data has been published from the United States (8) and the United Kingdom (3) regarding this pattern. Such data substantiates the many stories voiced by children, parents, and school staff: that more children are missing school more often than before the onset of the pandemic. A growing body of literature has identified mental health as a key factor within the school absenteeism discussion. These studies indicate that mental health challenges, generally, increase the likelihood of greater school absences for a child [e.g., (9)]. Additionally, there is now strong research evidence that demonstrates a clear link between the COVID-19 pandemic and deteriorating mental health for children and youth (10). While there is strong evidence that links mental health challenges with poorer school attendance concurrently, there is still little known about the association over time.

To address this gap in the literature, the present study provides clarity regarding the link between school absenteeism and mental health of students from kindergarten to Grade 12. In line with the transdiagnostic model of mental disorders (11), mental health will be categorized into externalizing and internalizing problems to reflect broader categories of challenges. The present study uses data from two separate timepoints to determine the longitudinal associations between mental health and school attendance patterns across a single school year in a sample of Canadian children and youth. T1 took place in Fall 2022, when students had returned to regular in-person schooling and T2 was in Spring 2023, when the COVID-19 pandemic was ending globally.

### Problematic absenteeism

Despite decades of literature investigating the frequency and types of school absenteeism experienced by students, there remains little to no consensus on what exactly constitutes problematic absenteeism. Problematic school absenteeism has proven difficult to define given the complexity and heterogeneity of its risk factors and presentation (12). Heyne et al. (13) summarise the literature on school attendance problems, describing the various ways it has historically been defined, including dichotomies, such as legitimate vs. illegitimate, and excused vs. unexcused absences. Such conceptualizations have been criticized and described as unrepresentative of students' experiences of school absenteeism (12, 14), failing to capture the nuance of why a student may not be present at school. Indeed, many instances of absenteeism are not so simple to dichotomize into one of the two groups. Consider a child a who is sick with a flu, but their parent does not have the capacity (e.g., working full-time) to acquire a doctor's note or call the school to excuse their absence. That child's absence will now be marked as unexcused by the school when in reality, it may be better conceptualized as an excused absence. This example illustrates the complexity of the problematic absenteeism issue and the need to acknowledge social and cultural systems surrounding the child. As well, despite a lack of quality evidence indicating a clear threshold of what constitutes problematic absenteeism, most countries and educational systems are adopting the benchmark of 10% of days missed or 18–19 days missed in the entire school year as constituting problematic (chronic or habitual) absenteeism [(15, 16); Ontario Educational Ministry]; the present study will also adopt this benchmark.

Beyond frequency of school days missed, reasons for days missed from school are also considered within a problematic absenteeism definition. One method of conceptualizing problematic school absenteeism is categorical. Heyne et al. (13) suggest problematic absenteeism can be categorized as *school refusal* (i.e., student refuses school and/or experiences distress while at school, parents are aware of absence and make attempts to bring their child to school), *truancy* (i.e., student absent from school without permission or parent knowledge), *school withdrawal* (i.e., student absent from school with parent knowledge and efforts to keep them home), and *school exclusion* (i.e., the school has unintentionally or intentionally reinforced or prompted absence from school). If an absence falls within one of these four categories, it is considered problematic. Unproblematic school absences are defined as being authorized/permissible [e.g., due to an appointment, being sick, weather-related (17)]. There is research that supports the categorization of problematic absenteeism in this way (13, 18, 19). However, it is worth noting that recent research has shown that any absence, whether labeled as problematic or not, can lead to poor outcomes later on in several domains [e.g., academic, cognitive, socioemotional (20)], including future absences (21, 22). As a result, the total amount of school absenteeism, regardless of its justification, is an important variable when considering student outcomes in various domains.

### Mental health and absenteeism

While absenteeism is a concern for all students, there is a particular worry for students who experience mental health challenges. A growing body of literature indicates that students who experience mental health and/or neurodevelopmental challenges (e.g., depression, anxiety, Attention-Deficit/Hyperactivity Disorder; ADHD) are at greater risk of missing school more frequently [e.g., (9, 23, 24)]. In a large-scale meta-analysis that included a total of 42 studies (*N* = 243 296 students) examining the link between school absenteeism and its risks, researchers found several mental health factors that were significantly associated with absenteeism rates [e.g., internalizing problems, psychiatric symptoms/disorders, depression, anxiety, and risky behaviour (25)].

Many diagnostic and conceptual labels can be given to a child or youth experiencing mental health concerns (e.g., anxious, depressed, conduct problems). As a way to simplify conceptualizations of mental health concerns, researchers and clinicians alike have proposed a transdiagnostic model of mental health that categorizes mental health concerns/symptoms into two broader clusters; that of externalizing concerns and of internalizing concerns (26). The model posits that categorical Diagnostic Statistical Manual – 5th Edition (DSM-5) diagnoses share common core elements that are often stable across time and promote a more comprehensive understanding of an individual's mental health profile (26). For example, both depressive and anxiety disorders share common symptoms: social withdrawal, nervousness, somatization, self-criticism. In this way, the transdiagnostic model considers mental health challenges more broadly and is not limited to labels that only capture some of the presenting symptoms. There is evidence to support that such a conceptualization of mental health can offer utility in understanding child and youth functioning and later outcomes (27, 28).

Recent attendance literature has adopted this transdiagnostic model of mental health in their analysis of school absenteeism. Rogers et al. (29) analyzed school absenteeism in relation to both internalizing and externalizing symptoms and found significant but differential associations with each—highlighting the importance of individually analyzing absenteeism across these two continuums. Specifically, Rogers et al. (29) found that that the association between internalizing behaviours and absenteeism was strengthened as children aged and as internalizing symptoms grew in intensity, while externalizing behaviours were found to be most strongly associated with school absenteeism for younger children with low to moderate levels of externalizing behaviours. Other studies in educational psychology have adopted this transdiagnostic conceptualization of mental health challenges. Abry et al. (30) analyzed the association between mental health concerns (e.g., categorized as internalizing or externalizing) and classroom level adversity (including absence rates) and found substantial and differential effects between these two groups of students. Specifically, classroom level adversity was significantly related to externalizing behaviour for all students, whereas classroom level adversity was related to internalizing behaviours only for girls. Given its descriptive utility, the present study also describes mental health concerns using the transdiagnostic model. Importantly, there is minimal longitudinal research on child mental health and school absenteeism. The present study seeks to fill this gap by examining the intersection of these two variables across time to determine their bidirectional or causal ordering.

### COVID-19 and child mental health

It is essential to situate the conversation of school attendance and especially child and youth mental health within the COVID-19 pandemic context. A recent large scale systematic review summarizing literature across five continents, indicated increased reports of anxiety, depression, and other mental health related challenges as a result of the COVID-19 lockdowns (31). Children and youth with pre-existing mental health challenges were more likely to experience an exacerbation of these challenges. As well, children and youth with previous mental health difficulties were more likely to experience a reoccurrence of their challenges.

New data published in Canada outlines the negative impacts of the COVID-19 pandemic for Canadian children and youth's mental health. For example, one study investigated outpatient mental health visits of children and youth in the 18 months following the onset of the pandemic. The study results indicated a 22% increase in new mental health visits, and a 10% increase in mental health visits of those already seeking care (32). Additionally, the Canadian Institute for Health Information (CIHI) reported that surveys amongst Canadian children and youth indicated a 20% decline in reports of good mental health and that Kids Help Phone reported double the number of interactions during the pandemic in 2020, compared to pre-pandemic in 2019 (33). Recent Statistics Canada data further bolsters concerns regarding Canadian children and youth's mental health with data showing that approximately 1 in 5 children and youth who reported good mental health prior to the pandemic no longer felt their mental health was good in 2023 (34). Taking both Canadian and international studies together, there are strong indications that child and youth mental health has declined since the onset of the COVID-19 pandemic. Such studies further highlight the need to longitudinally examine school absenteeism patterns for children struggling with mental health challenges given what is known about the concurrent links between mental health and school attendance problems and the widespread negative impacts of the pandemic on child and youth wellbeing.

### Current study

While other countries have mechanisms in place to systematically collect school attendance data (e.g., United States, UK), such data does not exist on a national level in Canada. Educational decisions are made provincially/territorially in Canada, which further complicates efforts for a unified approach to data collection. Pockets of Canadian school absenteeism data can be located but it is fractured. A recent CBC investigative article compiled attendance and absenteeism data from 41 Canadian school districts (1). Complete data was received from eight districts, two provinces, and two territories (the last through publicly available data). Twenty school districts were unable to provide data. Of the available data, estimates indicated significant increases in chronic absenteeism during and post pandemic. In some districts, approximately 50% of the students were considered chronically absent. The present study investigates school absenteeism among Canadian children and youth by assessing longitudinal data. Specifically, the current study examines school absenteeism across time, and how mental health challenges intersect with student absenteeism from Fall 2022 (as students returned to regular in-person schooling) to Spring 2023 (as COVID-19 pandemic was ending, globally). The research questions are:

1. Overall, how much school are children and youth missing? What are the most common reasons for absenteeism?
2. What is the magnitude and direction of the longitudinal links between mental health challenges (i.e., dichotomized as externalizing and internalizing) and problematic absenteeism?
3. What are the differences and similarities regarding the reasons for school absenteeism between students with externalizing mental health challenges and internalizing mental health challenges?

Given the lack of Canadian data available regarding the frequency and reasons for school absenteeism, the first research question is explorative in nature. Longitudinally, we expect that the links between mental health challenges, both externalizing and internalizing, and school absenteeism will be positive and bidirectional (i.e., a reciprocal loop between mental health difficulties and problematic absenteeism). Medium effect sizes are anticipated given that literature has documented effects ranging from small to large (23, 24). It is expected that the patterns linked to specific mental health concerns and type of absenteeism (e.g., students with elevated internalizing symptoms report greater frequency of school refusal as reason for school absence) will emerge.

## Methods

### Participants

The sample included a total of 72 children and youth (mean age = 12.28, *SD* = 3.1) for both timepoints of data. Age data was missing for 3 participants. Gender was evenly split between boys and girls. The youngest child represented in the data was just over 5 years at the time of parent reporting, while the oldest adolescent was 17 years. All grade levels were represented in the sample from kindergarten to Grade 12, with the greatest number of students in Grade 7. See Table 1 for a breakdown of all demographic information of the study sample.

**Table 1:** Demographic characteristics and mean absenteeism rates at T1 and T*2.*

Demographic grouping	Proportion at T1 (%)	Mean absenteeism rate in days (T1)	Mean absenteeism rate in days (T2)
Gender			
Boy	51.4	2.3	1.7
Girl	48.6	2.6	2.2
Race/Ethnicity			
White/Caucasian	86.1	2.4	2.0
Black	1.4	0.0	.5
Indigenous	12.5	2.6	2.1
Geographic Region			
British Columbia	25.0	2.5	2.3
Alberta	15.3	2.1	1.5
Saskatchewan	1.4	0.0	0.5
Manitoba	6.9	3.4	2.0
Ontario	36.1	2.5	1.9
Quebec	5.6	1.8	1.5
New Brunswick	1.4	4.0	0.0
Nova Scotia	2.8	1.5	2.0
Newfoundland and Labrador	1.4	3.0	2.0
Northwest Territories	2.8	3.0	2.5
Nunavut	1.4	2.0	4.0
Annual Household Income			
$6,999 or less - $32,999	5.6	2.8	2.8
$33,000 - $52.999	19.4	2.5	2.5
$53,000 - $105,999	59.7	2.6	2.1
$106,000 - $264,999	6.9	0.6	0.1
$265,000 or more	6.9	2.6	0.6
Highest Education Level			
High school or GED	–	–	–
Some college	8.3	1.8	2.2
College diploma	20.8	2.5	1.9
Bachelor's degree	48.6	2.9	2.0
Some graduate work	4.2	2.2	2.0
Master's degree	15.3	1.8	2.0
Doctoral degree	1.4	0.0	0.0
Number of Hours Worked			
0–5 h	–	–	–
6–20 h	1.4	1.0	2.0
21–39 h	70.8	2.6	2.2
40 h or more	27.8	2.1	1.4

The mean absenteeism rate in days at each timepoint reflects the 2-week time periods in which the parents reported on.

### Procedure

Ethics approval was received at the universities of the principal investigators (M.R. & Y.J.). The study data is taken from a larger research initiative querying the experiences of parents during and in the aftermath of the COVID-19 pandemic. Recruitment occurred through online recruitment tools and included online advertising through a variety of professional platforms including the Canadian ADHD Resource Alliance (CADDRA), and the Centre for ADHD Awareness (CADDAC). The psychology labs of the collaborating co-investigators also advertised amongst their child and family participant lists. The data for this study spans one academic year across two separate timepoints: Fall 2022 and Spring 2023. Parents who expressed interest in their continued participation from an initial timepoint were included in the present study.

Parents provided information about their child. Parents of children and youth were included if their child was reported to be in grades K-12, resided in Canada, and parent/guardian(s) were proficient in English and able to complete the survey in English. Parents were asked to report on the same child for each timepoint.

The survey was administered to parents via the Qualtrics online platform. Interested parents were asked to provide consent before proceeding to the survey, and were asked at the end of the survey to provide their consent for compensation. They were also asked if they wished to be contacted again for future timepoints/studies. Relevant to the present study, parents were asked to complete questionnaires that gathered demographic information, as well as details about their child's school attendance and mental health. The survey took approximately 30–45 min. Parents were asked to complete several other measures as part of the larger questionnaire, but these are outside the scope of the current work.

### Measures

#### Demographics

Parents were asked to answer several questions about their own demographic characteristics, as well as those of their child who they were reporting on. Included in the present study analysis are: child age, school grade of the child, gender identity of the child, race/ethnicity of the child, geographic region of the family, annual household income, highest level of education in the household, and the number of hours per week worked of the reporting parent.

#### Frequency and type of absenteeism

To determine the frequency of school days missed and the reasons for school absenteeism, parents completed a modified version of the School Non-Attendance ChecKlist [SNACK (13)]. Relevant to the present study, the questionnaire was modified to reduce the timeframe in which parents were asked to estimate their child's number of absences (from 4-weeks to 2-weeks) to improve reliability of parental responses. As well, additional reasons for school absenteeism were added in order to better capture the heterogeneous reasons that explain a child's absence from school. Additional reasons included: “My child attends school on a part-time or modified basis”, “I let my child stay home because I like having them around”, “I decided to keep my child home because I feel as though the school is not meeting my child's needs”, “I kept my child at home because I no longer have the energy to get them to school”, “My child stayed home for COVID-related reasons”, “My child attends appointments to support their mental health”, “My child did not feel welcome at school due to racial and/or identity discrimination”, “My child stayed home for menstruation related reasons”, and “My child missed school because of sleep related challenges”. These changes were made with permission from the original author of the measure and through consultation with experts in the field of child and youth mental health and wellbeing.

Parents were asked to report any absences that had occurred in the past two weeks and to identify the reasons why their child was absent from school on those days. Parents could select as many reasons from a list that apply to explain their child's school absence(s), since one absence may be explained by multiple reasons (e.g., child was reluctant and parent no longer had the energy to get them to school). Parents could also write their own reason if the reason for their child's absence was not represented in the provided list. In this way, the modified SNACK allows for systematic categorization of each absence into one of five categories: school refusal, truancy, school withdrawal, school exclusion, or non-problematic (e.g., absences that are excused, such as extreme weather conditions). The SNACK was chosen since it is one of the only absenteeism measures that assesses for the four different kinds of problematic absenteeism as well as non-problematic absenteeism, and inquires about the specific number of full and half days missed in the recent past. It is also a brief measure that takes little time to complete, which is important in consideration of the potential lack of time available from parents in the context of the pandemic. Given that the SNACK is count data, there is no psychometric data available in the relevant literature and traditional reliability and validity methods are not possible.

It is worth highlighting that there were minor modifications made to the attendance measure between the two timepoints. Most relevant to the current study is the item from T1 “My child did not feel welcome at school due to racial and/or identity discrimination” was changed to “My child did not feel welcome at school due to discrimination” in T2 to enlarge the definition of discrimination and allow for capturing other types of discrimination. As well, an additional reason for absenteeism was added in at T2, “My child stayed home due to bullying-related reasons” given interest in understanding absenteeism specific to bullying. These changes were made to reflect feedback from parents and mental health professionals working closely with children, youth, and families.

#### Child and adolescent symptom inventory (CASI-Pm)

The Child and Adolescent Symptom Inventory [CASI-PM (35)] is a 29-item scale that assesses various mental health symptomatology of children and youth aged 3–17 years of age. The scale assesses symptoms related to ADHD, oppositional defiant disorder, conduct disorder, generalized anxiety disorder, social anxiety disorder, separation anxiety, and major depressive disorder. Parents were asked to report the frequency with which their child had engaged in the behaviour described in each item using a 4-point Likert scale; from (0) *never,* (1) *sometimes,* (2) *often,* to (3) *very often.* The internal consistency of the CASI-PM has been found to be very good with a Cronbach's alpha of .92 (35). Additionally, the CASI-PM shows good criterion validity when compared against other similar measures such as the longform of the Child Symptom Inventory (CSI) (35).

For the purposes of this study, the various mental health symptoms assessed via the CASI-PM were divided into two overarching categories of internalizing symptoms and externalizing symptoms, based on the transdiagnostic theory of mental disorders (11, 26, 35, 36). The ADHD (inattention and hyperactivity), oppositional defiant disorder, and conduct disorder subscales (four in total) were summed to create a total externalizing score for each child and youth. In this way, a higher score on the externalizing subscale indicated greater number and severity of externalizing symptoms. The Cronbach's alpha at T1 for the externalizing subscale was good (*α* = .84), indicating strong internal reliability. At T2, the reliability for the externalizing subscale was lower, Cronbach's *α* *=* .68. Similarly, the generalized anxiety disorder, social anxiety disorder, separation anxiety disorder, and major depressive disorder subscales were summed to create an internalizing score for each child and youth (i.e., higher score indicated greater number and severity of internalizing symptoms). At T1, the internal reliability of this subscale was good, Cronbach's *α* = .83. However, at T2, the internal reliability was lower. Cronbach's *α* = .66.

To assess the school absenteeism reasons for children with elevated internalizing and externalizing behaviours separately, the CASI data was categorized to create two separate groups of children and youth. Using the T1 data, the mean of the externalizing data and the mean of the internalizing data was computed. Children and youth were categorized into the externalizing group if they had an externalizing score above the sample mean in the current study (*n* = 38). Likewise, children and youth were categorized into the internalizing group if they had an internalizing score above the sample mean (*n* = 37). Given this method, there was some overlap amongst children and youth regarding which symptom group they were placed into in that some children and youth were represented in both the externalizing and internalizing group (*n* = 30).

### Data preparation

The study data underwent two rounds of data cleaning to ensure data validity and to remove any bot data. All participant data went through round one filtering when initially recruited. At each subsequent timepoint, participant data was subject to a second round of data cleaning.

In round one data cleaning, data were removed if parents (1) took less than 10 min to complete the survey, (2) were located outside of Canada according to their IP address, and (3) indicated that their child was outside 3–18 years and 11 months old. For round two data cleaning, data were removed if parents violated two or more of the following criteria, (1) duplicate IP address, (2) duplicate email address, (3) having more than a 5-year (±2) difference between the age and grade of the child being reported on. Additionally, there were a few participants with substantially non-matching dates of birth (>1 year difference). These participant data were checked further and only kept if school grade only differed by +/- one year between timepoints.

Other measures were taken to ensure data validity and to identify bot data such as the collection of IP addresses, a re-captcha bot screening question, and a question asking parents to select the largest number out of a selection of options.

### Data analysis strategy

Analyses were completed using SPSS (v.29) and R (37). Descriptive and frequency analyses were conducted to determine how school attendance behaviour related to various demographic information (e.g., race/ethnicity, gender, age, annual family income). Frequency analyses were also conducted to determine the most common reasons for absenteeism in the study sample. A series of cross-lagged panel analyses were conducted to determine the size and direction of effects between study variables, including the exploration of bidirectional effects. Age and gender were entered into the cross-lagged panel analyses as covariates to control for their effects. Frequency analyses were also run to determine if systematic differences emerged between reasons for absenteeism amongst different sample groups (i.e., externalizing and internalizing behaviours).

## Results

### Frequency and reasons for absenteeism

Parents in T1 (Fall 2022) reported their children and youth missing a mean of 2.42 days of school (*SD* = 1.16) over the preceding two-week period. In T2 (Spring 2023), the mean number of school days missed was 1.95 (*SD* = 1.16), in the preceding two-week period. This result indicates that children and youth were missing roughly one day of school per week at each timepoint. Age was correlated with absenteeism rates at T1 (*r* = .25, *p* = .04) indicating that absenteeism rates increased as student age increased. This association was not found at T2. There was no significant effect of gender. See Table 1 for attendance data in relation to demographic information.

The most common reasons for school absenteeism across the whole sample were assessed. Frequency analyses were conducted for both timepoints separately to determine the reasons for why the sample missed school most often. See Table 2 for the top reasons for each time point.

**Table 2:** Most common absenteeism reasons across timepoint*s.*

Reasons for absenteeism	N	Percentage of sample (%)
T1		
COVID-related reasons	45	62.5
Skipped/truanted	43	59.7
Reluctant/avoided	37	51.4
Parent no longer has the energy	34	47.2
T2		
Reluctant/avoided	44	61.1
Skipped/truanted	28	38.9
Non-COVID related illness	26	36.1
Parent gave child the day off	22	30.6

*N* = 72 for both timepoints.

### Path analyses on mental health and school absenteeism rates

Cross-lagged panel analyses were conducted to determine the direction and magnitude of longitudinal links between mental health challenges (dichotomized as externalizing and internalizing behaviours) and rate of absenteeism. Two separate cross-lagged panel analyses were run to assess the individual links for externalizing and internalizing behaviours. To maximize power, all available data was considered in the path analyses and no data was deleted listwise or casewise.

#### Externalizing challenges and school absenteeism

**Figure 1:**
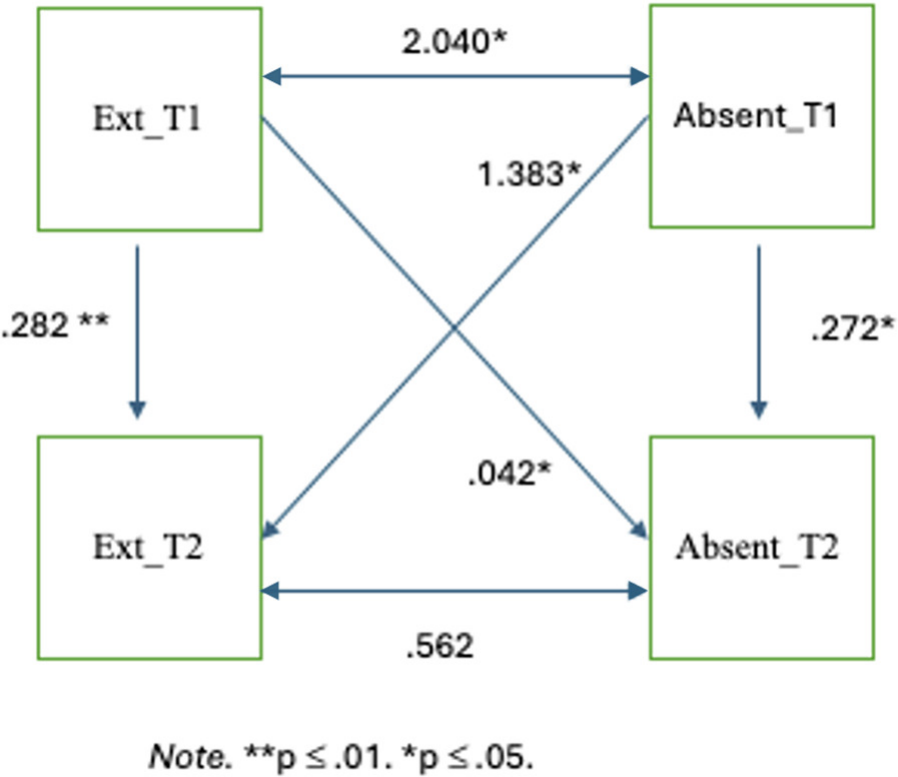
Externalizing behaviours and school absenteeism.

Externalizing behaviours (two timepoints), and rate of absenteeism (two timepoints) were added into the model. See Figure 1 for model and coefficients. Age and gender were included in the model as covariates and were held at zero. The Comparative Fit Index (CFI) was .88. The Root Mean Square Error of Approximation (RMSEA) was .08 [90% CI: (.00, .17)]. The overall model fit was also evaluated using the Chi-square statistic *χ*^2^(9, *N* = 72) = 13.00, *p* = .16. The Standardized Root Mean Square Residual (SRMR) was .08. These fit indices suggest a borderline acceptable fit with values sitting at or just below the acceptable range. The model showed that externalizing behaviours at T1 predicted externalizing behaviours at T2 (*β* = .28, *p* = .004), indicating that higher levels of externalizing behaviours at T1 were linked to higher levels of externalizing behaviours at T2. Additionally, absenteeism at T1 predicted absenteeism rates at T2 (*β* = .27, *p* = .02). Absenteeism at T1 significantly predicted higher levels of externalizing behaviours at T2 (*β* = 1.38, *p* = .02). The effect size was small to moderate. A bidirectional pathway was identified. Externalizing behaviours at T1 predicted increased rates of absenteeism at T2 (*β* = .04, *p* = .03). This effect size was also small to moderate. Additionally, the model indicated that externalizing behaviours and absenteeism rates at T1 significantly covaried with an estimate of 2.04, *p* = .03, suggesting that absenteeism rates and externalizing behaviours are significantly associated within the same timepoint. Specifically, elevated externalizing behaviours in Fall 2022 were linked to elevated school absences in the same timepoint. This relationship was not found at T2.

#### Internalizing challenges and school absenteeism

**Figure 2:**
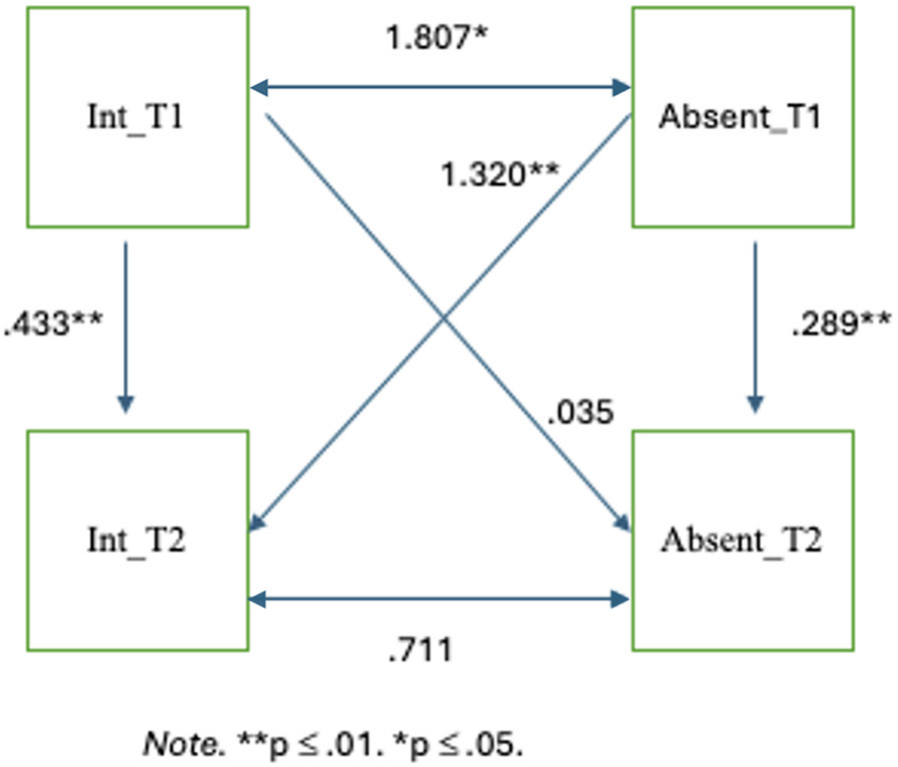
Internalizing behaviours and school absenteeism.

Internalizing behaviours (two timepoints) and rate of absenteeism (two timepoints) were added into the model. See Figure 2 for model and coefficients. As with the externalizing model, age and gender of the child were included as covariates and held at zero. The Comparative Fit Index (CFI) was .88. The Root Mean Square Error of Approximation (RMSEA) was .098 [90% CI: (.00, .18)]. The overall model fit was also evaluated using the Chi-square statistic, *χ*^2^(9, *N* = 72) = 15.20, *p* = .09. The SRMR was .097. These model indices indicate a borderline acceptable fit with values sitting just within or outside the acceptable/marginal cutoffs. The model showed that internalizing behaviours at T1 predicted internalizing behaviours at T2 (*β* = .43, *p* = .001). That is, higher levels of internalizing behaviours at T1 were predictive of higher levels of internalizing behaviours at T2. The model also showed that absenteeism rates at T1 were significantly predictive of absenteeism rates at T2 (*β* = .29, *p* = .01). Additionally, the model indicated that absenteeism rates at T1 significantly predicted internalizing behaviours at T2 (*β* = 1.32, *p* = .003) indicating that higher levels of school absenteeism in Fall 2022 predicted greater internalizing challenges in Spring 2023. This effect was small to moderate. No significant pathway was found between internalizing behaviours at T1 and absenteeism rates at T2. It is worth noting that absenteeism rates and internalizing behaviours at T1 covaried significantly with a covariance of 1.81, *p* = .02, indicating that absenteeism rates and internalizing behaviours are significantly associated within the same timepoint. This relationship was not found at T2.

### Most common absenteeism reasons for children with externalizing and internalizing problems

Frequency analyses were conducted to determine the most common reasons for missing school for students with elevated externalizing and internalizing problems at T1. See Table 3 for a summary of the four most common reasons for each mental health group.

**Table 3:** Most common absenteeism reasons for children exhibiting externalizing and internalizing behaviours at T1.

Reasons for absenteeism	N	Percentage of sub-sample (%)
Externalizing		
For COVID-related reasons	27	71.1%
Skipped/truanted	26	68.4%
Parent no longer has the energy	18	47.4%
Reluctant or avoided	18	47.4%
Internalizing		
Skipped/truanted	23	62.2%
For COVID-related reasons	22	59.5%
Reluctant or avoided	15	40.5%
Parent no longer has the energy	15	40.5%

There were 38 children in the externalizing group analysis and 37 children in the internalizing analysis.

## Discussion

The present study sought to provide a better understanding of the link between school absenteeism and mental health challenges within a sample of Canadian children and youth. This is the only study, to our knowledge, that examines the links between mental health and school absenteeism from a longitudinal, bidirectional lens using a Canadian sample. This study uses data collected within the COVID-19 pandemic context. The results provide important information regarding the links between mental health and school absenteeism that can inform interventions.

Students in our sample missed approximately one day of school per week over the two-week time periods. This estimate represents just one point in time and it is unknown what attendance patterns existed throughout the school year between T1 (Fall 2022) and T2 (Spring 2023). However, if we extrapolate these four weeks of attendance data to the entirety of the school year, then on average, the children within our sample would have missed a total of 37 days, which would fall well above the problematic absenteeism cutoff of 18–19 days [(15, 16); Ontario Educational Ministry]. While speculative, this frequency of school absenteeism demonstrates how quickly school absences can add up and that reaching the 18–19 day benchmark that is considered “problematic absenteeism” is not difficult. This finding may also speak to the impact of the COVID-19 pandemic in elevating absenteeism rates for students relative to pre-pandemic estimates, which aligns with previous literature examining the link between COVID-19 and school absenteeism (2). Of note, the most common reason for absences at the first timepoint, when children returned to regular in-person schooling, was for COVID-related reasons. Importantly, from a Canadian schooling perspective, the two timepoints were chosen to reflect general education time periods for students of all ages. Data collection began after one to two months of school to allow for a transitionary period of starting a new school year. Data collection at the end of the school year was completed before the last month of school, which is typically more flexible and less structured than previous months (e.g., field trips, school wide activities). Additionally, these time periods are not impacted by exam periods for secondary school students, which occur in January and June country wide. As such, data collection time periods were chosen to capture, as much as possible, the typical school year for students of all ages, without the possible confounding impacts of more or less demanding periods of time that could impact students' attendance patterns or mental health challenges.

Results also showed mixed findings regarding the links between absenteeism rates and age and gender. Age was significantly and positively correlated with absenteeism rates at T1 but this association was not found in T2. There was no significant effect of gender. Literature on the association between gender and school absenteeism is mixed with some research indicating gender differences exist [i.e., boys more likely to attend school regularly (38)], and others indicating no gender differences (39). As with gender, the literature on age and absenteeism is also mixed. Meta-analytic findings have suggested that rates of absenteeism increase as students age (25). However, other research supports the notion that absenteeism rates are stable throughout the schooling years (21), suggesting that early rates of absenteeism can predict future rates of absenteeism. Our study findings reflect the variability of these associations found in the absenteeism literature.

### Externalizing and internalizing symptoms and school absenteeism

We examined the direction and magnitude of the longitudinal links between mental health challenges (i.e., externalizing and internalizing behaviours separately) and absenteeism rates at two separate time points. Both models had a borderline acceptable fit with the study data, with fit indices falling within or just outside of the acceptable/marginal cutoffs. As such, interpretation of the model pathways was made with caution and in the context of theoretical considerations.

First, both externalizing and internalizing behaviours were stable across timepoints, with positive and medium effect sizes (.28 and .43 respectively). That is, children who exhibited either or both externalizing and internalizing behaviours at the start of the school year were likely to continue to experience these challenges at the end of the school year. This consistency is expected given the short duration of time between the two timepoints, indicating symptom stability within this timeframe. Previous studies that have assessed the trajectory of externalizing and internalizing behaviours across childhood and adolescence have found that symptomology often changes across time (40); however, such studies have timepoints that are multiple years apart, rather than months.

Second, externalizing and internalizing behaviours shared a small to moderate association with school absenteeism within timepoints. Only links at T1 were statistically significant for both externalizing and internalizing behaviours. These results illustrate that both externalizing and internalizing challenges are concurrently linked with higher rates of school absenteeism, corroborating a growing body of literature that there are meaningful associations between ongoing mental health concerns and school attendance (9, 29). It appears that regardless of the mental health challenge (e.g., anxiety, depression, conduct, impulsivity-related difficulties), these problems are strongly linked to simultaneous poor school attendance. This evidence indicates the importance of generally addressing school attendance problems in students who are experiencing mental health challenges. Identification of such students may be an important avenue for earlier prevention of school attendance problems later on.

#### Longitudinal findings

Perhaps the most notable findings of the present study are the longitudinal associations. In both models, absenteeism rates at T1 were predictive of externalizing and internalizing behaviours at T2. One significant bidirectional association was found whereby a significant causal link was identified between externalizing behaviours at T1 and absenteeism rates at T2, suggesting a positive reciprocal loop between externalizing behaviours and absenteeism rates, as hypothesized. However, no significant bidirectional pathway was found in the internalizing model, suggesting that absences may play a particularly important role in driving the link between internalizing mental health challenges and later school absenteeism.

Few studies have examined the bidirectional association between mental health challenges and school absenteeism. In fact, there is sparse literature examining school attendance at all from a longitudinal perspective. Two studies in particular have examined the bidirectional effects of mental health concerns and school absenteeism; these studies are over a decade apart and have disparate findings (41, 42). Contrary to what would be expected, Panayiotou et al. (41) found that increased emotional difficulties at one timepoint were linked to reduced absences at a later timepoint. They did not find any longitudinal associations indicating that increased absences led to increased emotional challenges. Conversely, Wood et al. (42) found evidence for bidirectional pathways, but somewhat greater support for mental health difficulties impacting absenteeism rates. The bidirectional results from the externalizing model of the present study provide support for Wood et al. (42) findings regarding the link between absenteeism and conduct problems specifically. That is, children and youth in our sample who presented with greater externalizing behaviours were more likely to experience higher rates of subsequent school absenteeism, and more frequent absences from school predicted later externalizing challenges. However, the results of the present study also differ in important ways from both of these studies and suggest different patterns of association for externalizing and internalizing mental health challenges as well the possible critical role of absenteeism rate in future internalizing mental health challenges for the study sample.

The concerning finding that absenteeism appears to be a risk factor for future poorer mental health highlights the importance of school attendance for reasons far beyond academic functioning and achievement. Our results suggest that when a student does not attend school regularly, they are more likely to experience deteriorating mental health. One possible reason for this finding is that school absenteeism may exacerbate already underlying factors that may not be fully present in the child or youth's life, particularly in regard to internalizing mental health challenges. For example, consider a child who feels anxious much of the time but is still able to attend school, spend time with friends, and meet daily expectations. However, they become sick, perhaps with COVID-19, and miss an entire week of school recovering. Perhaps the thought of going back after having missed so much school elevates their anxiety and worry about what others might think.

### Reasons for absences

Descriptive analyses were conducted to examine the various reasons for school absenteeism amongst the total sample of children, and for those experiencing elevated externalizing and internalizing challenges separately. It is worth remembering that T1 data collection occurred during Fall 2022 (when students were attending regular in-person education again) and T2 was in Spring 2023 (just before the WHO officially declared that the COVID-19 pandemic was no longer considered a global health emergency). Additionally, given the lack of research on the reasons for school absences, this analysis and discussion are purely exploratory and therefore meant to paint a more descriptive picture of why the present sample of children were not in school.

In both timepoints, children in the total sample were most likely to be absent from school because they were sick (either from COVID-19 in T1 or another illness in T2), because they skipped or truanted, or because they refused school. Such findings demonstrate a stability in absenteeism reasons across the school year for the whole sample. These findings also speak to a wide range of factors influencing school absenteeism and can be mapped onto most of Heyne et al. (13) absenteeism categories. For many of the students in our sample, their absences would have been considered “non-problematic” given that they missed for illness-related reasons. In previous absenteeism literature, this would be classified as an excusable absence (43). Many other parents identified school refusal and skipping school as primary reasons for school absenteeism. These two reasons speak to two very different motivations and drivers of school absenteeism. Children and youth who refuse school tend to do so for anxiety-related reasons and with parent knowledge; however, children and youth who are truant are considered to do so without parental knowledge (e.g., arrive at school and then leave the premises). Interestingly, parent reports of “I no longer have the energy” was of the top reasons given for absenteeism in T1, while “I gave my child the day off” was a primary reason for T2. These two reasons, although different, perhaps speak to a common underlying thread. That is, in both instances parents know that their child is not attending school and may be capitulating to or supporting the child's or their own desires to not attend. Of course, these reasons also differ in important ways. Primarily, “I no longer have the energy” suggests a parent who desperately wants their child to attend school, has likely tried to have their child attend school many times over, but has given up. “I gave my child the day off” speaks more to a parent who may not value school attendance over other priorities, such as a mental health day, or family activities. This parent may also let their child stay home so that both the parent and child can have a rest. Importantly, these two reasons highlight the integral role of the parent in a child's school attendance.

When looking to the reasons for school absenteeism for students presenting with elevated externalizing and internalizing behaviours, the top four reasons are the same. Overall, the overlap of reasons may speak to a shared experience for parent and child, despite different presenting mental health problems. Although speculative, mental health concerns seem to exhaust parental support, and may exacerbate the amount of school children miss. Aligning with previous literature, findings indicated that children and youth with elevated externalizing challenges were more likely to be truant (44), compared to the children and youth in the internalizing challenges group. Research has shown that children with higher levels of conduct problems (subsumed under the externalizing category) are also more likely to experience school exclusion (e.g., asked to leave early, suspended) (45). However, this did not reveal itself to be a primary reason for school absenteeism for our study sample.

It is also notable that for both the externalizing and internalizing group, the most common reason for school absenteeism at T1 was a COVID-19 related reason, suggesting that it was perhaps the unique impacts of COVID-19 that were driving the link with poorer mental health at T2. For example, a child contracts COVID-19 and now must not only miss school for several days, they must also isolate from all other activities and wear a mask when going out in public. This child may indeed wish to attend school and cannot—missing out on academic activities, social interactions, and important events occurring at school. This child may begin to experience feelings of anxiety (e.g., “It has been so long, what will it be like to come back?”), depression (e.g., “I feel so lonely”) and perhaps other externalizing challenges (e.g., acting out because they are irritable or bored). This finding helps to explain the complex interplay of school attendance and mental health challenges within the specific context of the COVID-19 pandemic, which may be the lynchpin to understanding the present study results regarding the link between mental health and school absenteeism because of its unique and negative impacts on children's schooling and wellbeing. It also provides support for the notion that any reason for missing school is problematic and can lead to adverse negative consequences. In the specific case of COVID-19, many children stayed home from school for excusable and valid reasons, and yet, these same children were also more likely to experience poorer mental health in subsequent months.

As with the absenteeism reasons for the whole sample, the reasons for each of the externalizing and internalizing groups indicate a wide range of reasons for missing school that can be divided into Heyne's (13) categories for school absenteeism: school refusal, truancy, school withdrawal, and non-problematic absenteeism, if any absenteeism can be considered non-problematic. These results provide further support for Heyne's model of organizing school absences in this way and highlight the need to inquire about reasons for absenteeism as this will determine different intervention approaches. This data around reasons for absenteeism is helpful in providing a more comprehensive picture of the absenteeism patterns of students. The reasons behind why students miss school is largely missing from the literature, and inevitably inhibits our ability to effectively intervene and support students towards regular school attendance.

### Limitations and areas for future research

While the present study makes an important contribution to the field of school attendance research, this study is not without its limitations. First, this project relied on convenience sampling for participant recruitment. This recruitment strategy no doubt has important consequences regarding who might be captured in our sample and must be considered when interpreting the results. School absenteeism is a sensitive topic that is often linked to feelings of shame and guilt for parents. Despite efforts to reduce shame and stigma around school absenteeism in the questionnaire items, it is possible that some families opted to not participate given their emotional experience around school absenteeism. As well, families who chose to participate in the study had the time and energy in their day to complete a relatively large questionnaire (and across multiple timepoints). Families with parents/caregivers who work multiple jobs, for example, may not have had time or resources to complete the study questionnaire. It will be important for future research to collect data from Canadian families that is more reflective of the diversity of the population in all demographic regards. Doing so will provide even more comprehensive of an understanding of school attendance for Canadian children and youth generally.

Additionally, in relation to convenience sampling and anonymous, virtual data collection, there is the possibility for error within the data. As discussed, considerable efforts were made to minimize any data errors and remove any possible bot data. However, it is possible that given the method of data collection, error within the data remains. Interpretations of the study findings must be made with this consideration in mind and future research on the topic should strive to collect data through other methodologies that further minimize error in reporting.

Second, the sample size of the current study was relatively small and therefore limited statistical power, which may be a key reason for the poorer model fit (46, 47). This also limited our ability to make confident interpretations of the data and restricts our ability to generalize study findings beyond the current study sample. Larger sample sizes that increase power will allow for even more complex longitudinal designs that control for important confounding variables and may offer greater ability to generalize to wider populations. For example, larger samples would allow for analyses that consider developmental and age-related factors, such as shifting school experiences and responsibilities, which we were not able to examine within the current study. This is an important next step for future research to consider in the absenteeism literature, given that our study findings demonstrate age could be an important correlate when examining school absenteeism.

Additionally, the present study only included two timepoints for data analysis. While this still permitted the longitudinal investigation between the study variables, it does restrict our ability to determine causal ordering across time. It is possible that with an additional timepoint, differential longitudinal pathways would have emerged that would have improved the clarity of the interplay between mental health and school absenteeism across the entirety of the school year. It is recommended that researchers examining school attendance make efforts to conduct longitudinal research across multiple timepoints. It will also be important to examine the links between mental health and school absenteeism across schooling years, to better understand the size and direction of the links across a substantial period of time, rather than just within a single school year.

Our study also raised important considerations worth investigation in future research. First, we recommend that future research examine the effect of time of year that data collection occurs, which can impact time spent in outdoor activities and daylight exposure. These factors have known impacts for mental health challenges, and in particular mood-related disorders (48), whereby more outdoor activity and exposure to daylight has been found to have positive impacts for child and youth mental health through interactions with sleep duration. Although this was outside of the scope of the current study, we posit that these factors are worth consideration, particularly from a Canadian context, given that Canadian winters typically span several months with high snowfall, cold temperatures, and short periods of daylight, inhibiting opportunities for both outdoor activities and sunlight exposure. Research regarding these factors in relation to school attendance may provide useful insights into how and when to support children in attending school regularly.

Lastly, it is possible that somatic symptoms associated with mental health challenges of the children and youth within our sample were conflated for illness-related absences. It is well documented that mental health challenges such as anxiety and depression are linked to uncomfortable and distressing bodily sensations [e.g., headaches, stomach aches, low energy (49);]. This is a limitation of the present study and it is recommended that future researchers attempt to parse out the impacts of somatic symptoms related to mental health challenges when assessing school attendance as this has important intervention implications for children and youth.

### Implications and applications for intervention

The present study adds to the small body of literature that investigates school absenteeism and mental health across time. The results bolster Heyne's (13) conceptualization of the categorization of school absenteeism reasons and substantiate previous literature findings demonstrating a link between mental health challenges and school absenteeism. To our knowledge, there appears to only be two previous studies that have examined mental health and school absenteeism bidirectionally (41, 42). The present study's results do not align with previous literature in important ways, thus highlighting the complexity of links between school attendance and mental health, as well as the fundamental need for more research on the topic.

Importantly, there is very little Canadian data regarding school absenteeism (50). This lack of research appears to be a systemic problem, in that there are virtually no educational researchers in Canadian university institutions that primarily study school absenteeism (51). The present study seeks to contribute to the Canadian knowledge landscape of school absenteeism, and the intersections with mental health. This study also provides essential information for those working with children, youth, and their families around school attendance issues. The results speak to an urgent need to address school attendance problems, to intervene against possible subsequent mental health challenges. Given the bidirectional associations between externalizing behaviours and absenteeism, children exhibiting externalizing behaviours should be identified and monitored in order to intervene in a timely manner so as to avoid future school absences. The results also highlight that any absence, even those that are “excusable”, may remain detrimental to a child's wellbeing and future functioning. It is recommended that individuals who work closely with children and youth monitor the frequency of school absences, no matter the reasons provided. In this way, there can be more timely intervention to reduce the total number of absences and decrease the likelihood of later mental health challenges.

The results affirm interventions such as the Multi-Tiered Systems of Support Approach [MTSS (52);] which organizes various interventions to remediate school attendance problems into three tiers of increasing severity. This approach encourages an individualized approach to supporting children and youth in their school attendance, given that children can miss school for a variety of different reasons, as our study results indicate. This approach also necessitates partnerships between home, school, and community organizations to collaborate around the issue and find the best-fitting supports to address the obstacles to a child's attendance at school.

## Conclusion

Together, the present study longitudinally examined the links between mental health and school absenteeism rates within a Canadian and COVID-19 pandemic context. Results indicated strong within timepoint associations between mental health and school absenteeism. The results also revealed that school absenteeism early in one school year was linked to increased internalizing and externalizing symptoms at the end of that school year. One bidirectional association was found whereby externalizing behaviours predicted later absenteeism. The present study adds to the growing body of literature investigating school absenteeism and mental health across time and bidirectionally, and is among the only research studies to examine school absenteeism in a Canadian context. It is hoped that this study encourages other researchers to focus efforts and resources on further expanding our understanding of school absenteeism among children and youth, as well as its correlates.

## Data Availability

The datasets presented in this article are not readily available because this data is not available publicly. Requests to access the datasets should be directed to Amanda Krause, akrau101@uottawa.ca.
